# Schrödinger’s Penguin? Divergent population trajectories and the challenge of assessing the threat status of the Erect-crested penguin

**DOI:** 10.1371/journal.pone.0359075

**Published:** 2026-10-21

**Authors:** Thomas Mattern, Klemens Pütz, Hannah Mattern, Robin Long, Dave Houston, Bianca Keys, Jeff White, Richard Seed, Myrene Otis, Ursula Ellenberg, Pablo Garcia-Borboroglu, Benno Lüthi

**Affiliations:** 1 The Tawaki Project, Dunedin, New Zealand; 2 Global Penguin Society, Puerto Madryn, Chubut, Argentina; 3 Department of Zoology, University of Otago, Dunedin, New Zealand; 4 Antarctic Research Trust, Zürich, Switzerland; 5 West Coast Penguin Trust, Hokitika, New Zealand; 6 Department of Conservation, Auckland, New Zealand; 7 Department of Marine Science, University of Otago, Dunedin, New Zealand; 8 Department of Public and Ecosystem Health, Cornell University College of Veterinary Medicine, Ithaca, United States of America; 9 Centro para el Estudio de Sistemas Marinos (CESIMAR-CONICET), Puerto Madryn, Chubut, Argentina; MARE – Marine and Environmental Sciences Centre, PORTUGAL

## Abstract

“Schrödinger’s Cat” is a quantum mechanics thought experiment in which a cat in a sealed box is considered both alive and dead until the box is opened and its true state observed. In species threat assessments, the reverse paradox can apply. We present the case of the Erect-crested penguin (*Eudyptes sclateri*), one of four extant penguin species currently listed as “Endangered” on the IUCN Red List – a classification based largely on limited and inconsistent observations from the 1970s. Between 2022 and 2024, we surveyed the entire global population of Erect-crested penguins, a species confined to two subantarctic island groups in the New Zealand region: the Bounty Islands and the Antipodes Islands. Population estimates were derived from drone surveys of the entire global range, conducted primarily during the incubation stage, complemented with ground counts to establish nest-to-bird ratios and estimate breeding population size. On the small Bounty Islands (~50 ha), we found approximately 27,800 breeding pairs – nearly as many as on the Antipodes Islands (~29,800 pairs), which are roughly 40 times larger and historically considered the species’ stronghold. Although data since the mid-1990s are sparse, available evidence suggests divergent population trajectories: the Bounty Islands population appears stable over at least the past 25 years, whereas the Antipodes Islands population has declined by more than 40% since the mid-1990s, a trajectory that approaches – and under precautionary assessment likely warrants – “Endangered” classification. These contrasting trajectories likely reflect fundamental differences between the two sites: on the Bounty Islands, birds have access to potentially more productive subtropical waters during the non-breeding season, while on the Antipodes Islands, Subantarctic skua predation on fragmenting colonies generates a self-reinforcing Allee effect and penguins face potentially less favourable oceanic conditions. Thus, like Schrödinger’s cat, the Erect-crested penguin population occurs in two states – stable and moving towards extinction – which only became clear once the proverbial box was opened. This situation highlights the challenges of assessing extinction risk when a species comprises spatially segregated populations on distinct demographic trajectories. While the Antipodes population clearly warrants concern, the stability of the Bounty Islands population would likely reduce the species’ global conservation status to “Vulnerable” under IUCN criteria. This creates both a communicative and conservation conundrum: the binary population trajectories – one stable, one declining – combine to improve the global threat category while effectively obscuring a severe and genuine population-level crisis, and may even weaken the fisheries protections critical to safeguarding what may be the species’ last stable stronghold, the Bounty Islands.

## Introduction

The world has entered an era of biodiversity loss with modern extinction rates up to 100 times above natural background levels, signalling the onset of the “sixth mass extinction” [1–3]. The global benchmark for assessing species’ conservation status is the IUCN Red List of Threatened Species (www.iucnredlist.org), which classifies species into categories ranging from “Least Concern” to “Critically Endangered,” with “Vulnerable,” “Endangered,” and “Critically Endangered” collectively recognised as threatened [4]. These categories are determined using a standardised set of quantitative criteria that evaluate extinction risk, with population size and its rate of change forming the core of the assessment, alongside measures of geographic range, and habitat extent and quality. The Red List’s strength lies in its ability to distil complex biological data into a standardised, globally recognised framework that can be readily understood and applied by decision-makers. However, because assessments are conducted at the global species level, the resulting category is, in effect, an aggregate status across all populations. This can mask the fact that some populations may be faring worse – or better – than the global listing suggests, a possibility highlighted in the IUCN Red List guidelines: “[...] taxa classified as Least Concern globally might be Critically Endangered within a particular region where numbers are very small or declining […]” [5].

Divergent trends in species population trajectories between regions can be a result of changing environments but also stem from uneven and/or unequal research attention or conservation investment across a species’ range. Furthermore, populations may be subject to varying degrees of anthropogenic pressure. For example, a numerically stable population within an active industrial zone could in principle face greater long-term risk than a declining population in a relatively undisturbed environment. Yet global averaging fails to account for such differences in site-specific pressures and risk. As a consequence, species with spatially divergent population trajectories may be assigned conservation statuses that underestimate localized extinction risk, despite ongoing population collapse in parts of their range.

In instances where a species’ distribution is limited to only a few locations, this can create an inverse parallel to the “Schrödinger’s Cat” thought experiment, which describes a cat in a sealed box that is considered both alive and dead until the box is opened and its true state observed [6]. While Schrödinger’s Cat exists in two contradictory states until its true fate is examined closely by opening the box, doing the same for a species may actually reveal that it exists in two contradictory states – declining at one site and stable or improving at another [e.g., 7]. This disconnect remains conveniently hidden within the “closed box” of outdated or aggregated global assessments, only becoming apparent when high-resolution, site-specific data are finally examined.

The Erect-crested penguin (*Eudyptes sclateri*) appears to provide a compelling example of such a mismatch between threat ranking and scientific evidence. Confined to only two subantarctic island groups – the Bounty Islands and the Antipodes Islands – located ~800 km southeast of New Zealand’s South Island and separated from each other by approximately 200 km of open ocean (Fig 1), the species is the least studied of all penguin species [8]. After a comprehensive description of the species’ anatomy and basic biology in the 1970s [9], it took 50 years before the first study of the Erect-crested penguin ecology was published [10]. In the intervening years, most information focused on estimates of population size with varying levels of accuracy, comparability and reliability [8,11]. The first published estimates of the world-population of Erect-crested penguins are based on surveys conducted by two concurrent expeditions to each of the archipelagos in 1978 [12,13], that estimated 115,000 breeding pairs on each of the Bounty Islands and Antipodes Island group, respectively. Although the validity of these estimates has since been questioned [14], the numbers nevertheless represent the foundation of the species’ current red list ranking [10].

**Fig 1:**
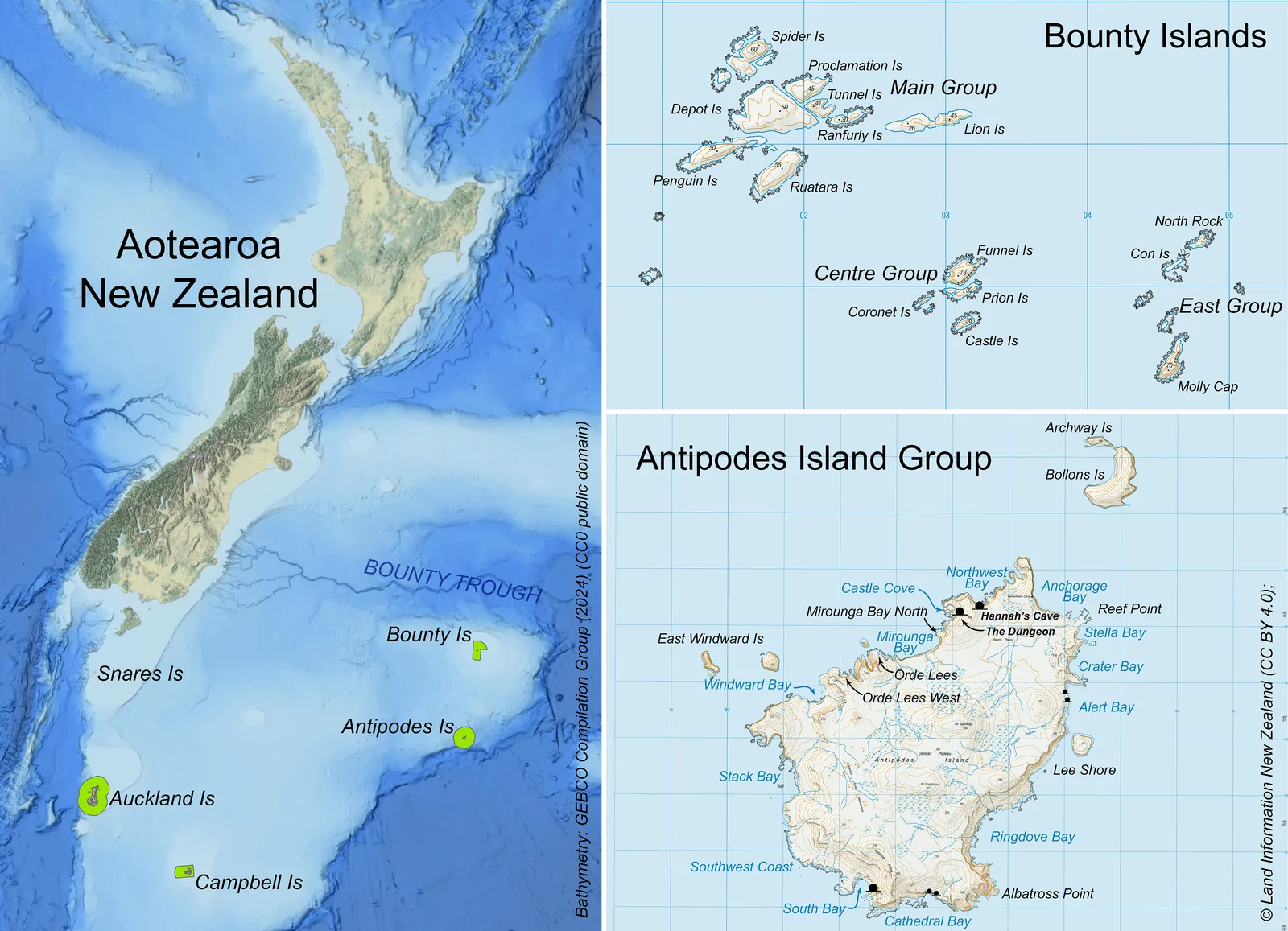
Location of Erect-crested penguin breeding archipelagos in the New Zealand subantarctic. The overview map (left panel) indicates the location of NZ’s five subantarctic island groups with marine reserves (green polygons) encompassing 100% of the territorial waters (12 nautical miles offshore) around the Auckland and Antipodes Islands, but only 58% around the Bounty Islands and 39% around Campbell Island. Bathymetry: GEBCO Compilation Group (2024) GEBCO 2024 Grid (https://doi.org/10.5285/1c44ce99-0a0d-5f4f-e063-7086abc0ea0f), released under a CC0 public domain dedication. LINZ Topographic map data are licensed under CC BY 4.0.

A review of four ground counts conducted on Proclamation Island in the Bounty archipelago between 1997 and 2019 found no significant change in the number of Erect-crested penguins during that time period [14]. In contrast, ground counts conducted between 1995 and 2014 at the species’ other stronghold, the Antipodes Islands, consistently documented a sustained downward trend, with a loss of about one-third of the penguin population over the course of two decades [10]. So despite an empirical base that is fragmentary in both spatial cover and temporal frequency, it appears as if the Erect-crested penguin might exist in two distinct population states – one stable and one in decline towards local extinction.

To resolve this “Schrödinger’s Penguin” dilemma, it is necessary to move beyond sparse site-specific counts and outdated estimates. The aim of this study was therefore to conduct a comprehensive, range-wide survey of the global population of Erect-crested penguins across both archipelagos, establishing a reliable and reproducible contemporary population benchmark, and to use these data in combination with previous estimates to derive valid population projections. We discuss results in the context of the species’ current IUCN Red List ranking, both when applied to the global population as well as to the two distinct subpopulations, demonstrating how aggregation into a single global ‘headline’ status can obscure divergent demographic trajectories and mask ongoing localised population collapse.

## Materials and methods

### Ethics statement

This study was conducted under a Wildlife Authority (86101-FAU), granted by the New Zealand Department of Conservation, which authorised both entry to the Bounty Islands and Antipodes Islands Nature Reserves and all interactions with wildlife during the course of this research. All procedures involving wildlife were additionally reviewed and approved by the University of Otago Animal Ethics Committee (AUP-21-43).

### Study sites

#### Bounty Islands.

The Bounty Islands comprise 14 islands, 11 islets and rocks, with a total landmass of 49.5 hectares [15] (Fig 1). First mapped by Captain William Bligh on 9 September 1788 and named after his vessel HMS Bounty [16], these granite islands rise sharply from sea level to heights of 40–60 m. Despite their barren nature, they support a recovering population of New Zealand fur seals (*Arctocephalus forsteri*) [17] and serve as breeding grounds for several seabird species, including Salvin’s albatross (*Thalassarche salvini*), Fulmar prion (*Pachyptila crassirostris*), and the endemic Bounty Island shag *(Leucocarbo ranfurlyi*) [12,18]. Erect-crested penguins have been recorded on 12 of the 14 main islands [19]. Due to their remoteness and harsh conditions, the Bounty Islands have been subject to only a handful of scientific expeditions over the past 50 years [13].

#### Antipodes Islands.

Situated approximately 200 km south of the Bounty Islands, the Antipodes Islands comprise the main Antipodes Island and several smaller satellite islands (Fig 1). Volcanic in origin, the islands cover approximately 2,100 hectares – roughly 40 times the area of the Bounty Islands (https://data.linz.govt.nz/layer/53564-protected-areas/). The terrain features rolling tussock grasslands and shrublands overlying a thick peaty topsoil [20]. The islands support 21 recorded breeding seabird species, including Erect-crested and Eastern Rockhopper penguins (*Eudyptes filholi*), Subantarctic skua/Hākoakoa (*Stercorarius antarcticus*), four albatross species, various petrels [21], as well as a range of terrestrial birds including endemic parakeets and resident passerines, whose populations have shown strong recovery following the successful eradication of invasive house mice (*Mus musculus*) in 2016 [22]. Along the coast, New Zealand fur seals are gradually recovering after near-extirpation due to historical sealing [13], and a small breeding population of southern elephant seals (*Mirounga leonina*) is also present at four sites [23]. Erect-crested penguins breed around the main island’s coast and on the East Windward, Bollons, and Archway Islands.

### Study species

The Erect-crested penguin (*Eudyptes sclateri*), weighing around 4–5 kg [8], is the largest of the seven recognized *Eudyptes* species [24]. On the Bounty Islands, these penguins breed in large mixed colonies alongside Salvin’s albatross (*Thalassarche salvini*), which can dominate available island surfaces [14]. In contrast, on the Antipodes Islands, Erect-crested penguins predominantly form mono-specific colonies situated in open areas such as rock platforms or tussock clearings near the coast (Fig 2). Occasionally, Eastern Rockhopper penguins breed alongside the species, though these smaller penguins typically nest in separate clusters or on the periphery of Erect-crested penguin colonies. A few colonies are found in sea caves [23].

**Fig 2:**
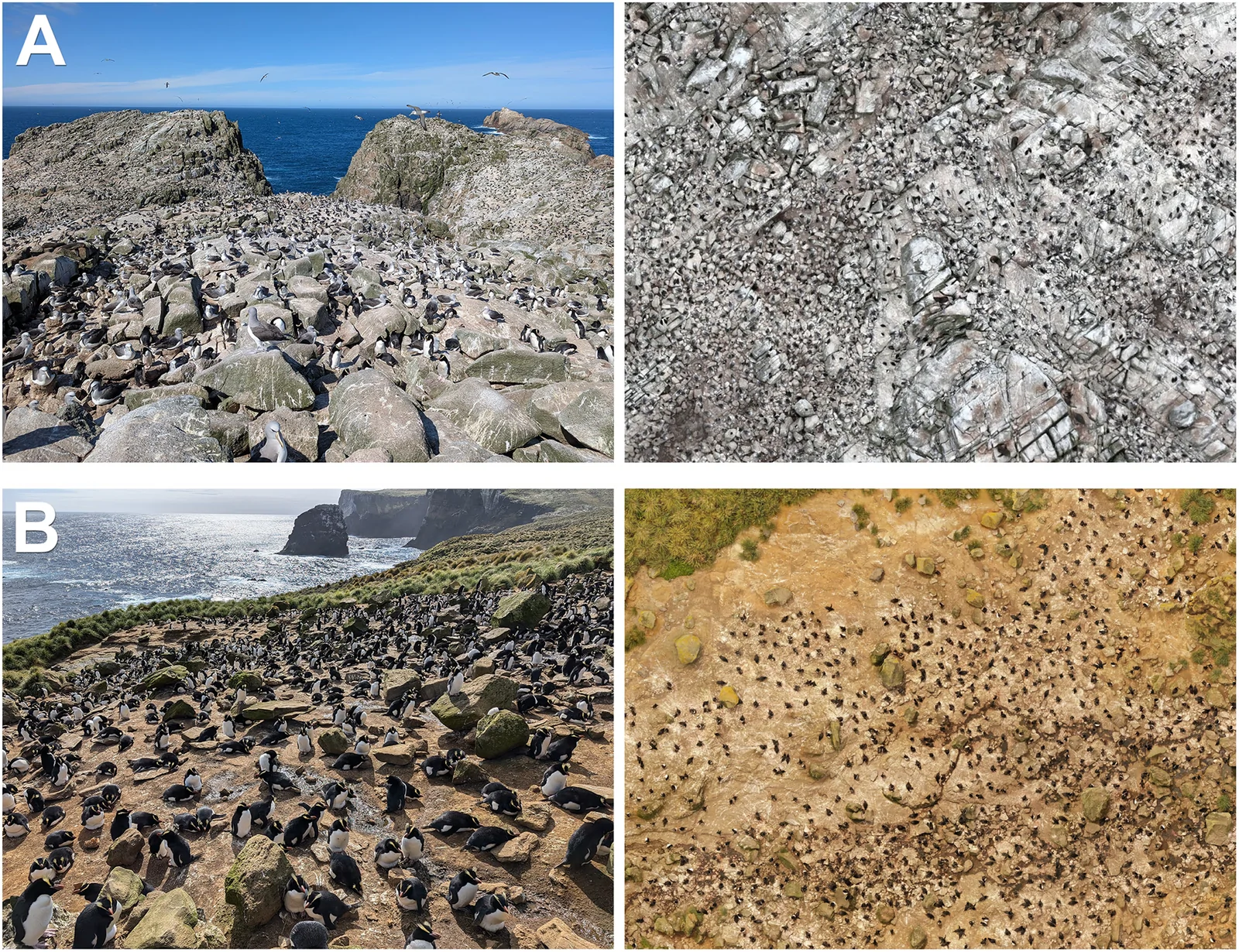
Contrasting breeding habitats of Erect-crested penguins on the Bounty Islands and the Antipodes Island group. (A) mixed colony of Salvin’s albatross and Erect-crested penguins on Depot Island, Bounty Islands, showing the bare granite substrate devoid of vegetation or soil; corresponding orthomosaic excerpt shown at right. (B) single-species colony at Stack Bay, Antipodes Island, on rock platform habitat denuded of vegetation by penguin activity; corresponding orthomosaic excerpt shown at right.

Breeding in Erect-crested penguins, similar to other crested penguins [25], follows distinct phenological stages. After pair reunion, eggs are laid in the first half of October on the Antipodes Islands and two to three weeks later on the Bounty Islands [8]. Both sexes remain at the nest for about two weeks post-laying, after which males embark on extended foraging trips. They return 1–2 weeks before hatching to relieve the females, who then undertake foraging trips of approximately one week [23]. Consequently, a single bird guards the nest for most of the incubation period. Following hatching, males guard the chicks while females make short one to two-day foraging trips to provision the offspring. Around three weeks post-hatching, males depart to forage for the first time in over a month, and chicks form crèches as organised nesting structure dissolves, before fledging in January and February [26].

### Timing of the surveys

Survey data on Erect-crested penguins presented in this study were collected during three expeditions in 2022, 2023, and 2024 [19,23,26]. The timing of these expeditions varied across years, capturing different stages of the penguins’ breeding cycle (Tables 1 and 2). Surveys conducted during the final three weeks of egg incubation provide the most reliable data for estimating breeding population size, as each nest is attended by a single breeding adult while the other is at sea [27].

**Table 1:** Summary of drone counts of Erect-crested penguins on the Bounty Islands, New Zealand, November 2022. Penguin numbers were determined by manual annotation of orthomosaics generated from drone surveys of the main islands in the archipelago. Nest numbers were estimated by applying a correction factor of 0.863 to penguin counts; this factor was derived from the ratio of ground-counted nests to annotated penguins on Proclamation Island (see results). No nest estimate is provided for Prion Island, where all birds present were identified as roosting without associated nests. Penguin distribution extent was quantified using the 90% kernel density contour, which was also used to calculate breeding area size and nest density; nest densities were calculated in all cases as estimated nests divided by breeding area. Totals for area and nest density exclude Prion Island; the penguin total includes the seven roosting birds on Prion Island. Total nest numbers were derived by applying the correction factor to the summed penguin count of all islands with nests and may differ slightly from the sum of rounded island values. Total nest density was calculated as total estimated nests divided by total breeding area.

Island	Area (m²)	Penguins (n)	Estimated nests (n)	Nest density (nests/m²)	Survey date
Depot Is	74,079	17,502	15,107	0.20	14 Nov
Ruatara Is	18,588	3,273	2,825	0.15	11 Nov
Proclamation Is	17,835	3,216	2,776	0.16	11 Nov
Funnel Is	12,033	2,761	2,383	0.20	14 Nov
Tunnel Is	10,206	2,153	1,858	0.18	10 Nov
Spider Is	23,357	1,736	1,498	0.06	10 Nov
Penguin Is	12,806	896	773	0.06	11 Nov
Molly Cap	6,292	257	222	0.04	15 Nov
North Rock	1,097	251	217	0.20	15 Nov
Ranfurly Is	2,651	83	72	0.03	10 Nov
Lion Is	1,367	64	55	0.04	11 Nov
Prion Is	258	7	–	–	14 Nov
**Total**	**180,311**	**32,199**	**27,788**	**0.15**	**–**

During the 2022 expedition, the Bounty Islands were surveyed during incubation [19], while surveys on the Antipodes Islands only coincided with this stage during the 2024 expedition [23]. The 2023 expedition occurred late in the breeding cycle during the crèche stage, when chicks move freely within colonies and can no longer be associated with specific nests. Furthermore, the number of breeding adults present in colonies fluctuates considerably throughout and between days at this stage [26], reducing the effectiveness of aerial counts for estimating the breeding population.

Penguin numbers were determined via aerial imagery recorded by drones and ground-truthing counts conducted by observers directly within colonies.

### Drone Counts

#### Aerial surveys.

Erect-crested penguin numbers were determined via drone surveys mapping entire breeding areas – and in the case of the Bounty Islands, entire islands.

Surveys were conducted using DJI Mavic 2 Pro drones (Shenzhen DJI Sciences and Technologies, Nanshan, Shenzhen, China) equipped with L1D-20c Hasselblad cameras featuring a fixed 28 mm-equivalent lens with an approximate field of view of 77°, capable of recording 20-megapixel still images. Using the mission planning app DroneDeploy (https://www.dronedeploy.com/), drones followed pre-defined zig-zag flight paths, capturing nadir images with 75% overlap between adjacent frames.

On the Bounty Islands, the entire archipelago was surveyed over four days – 10, 11, 14, and 15 November 2022; surveys were suspended on 13 November due to bad weather. Flight altitudes ranged between 40 and 60 meters above the islands’ highest points, depending on several factors: avoiding a layer of airborne albatrosses circling above the islands, accounting for the higher elevations of some islands, and minimising mission times for islands further from the launch site. All missions covering the main and central island groups were launched from the top of Proclamation Island, while surveys of the East Group islands were conducted from the deck of the research vessel.

The rugged topography of Antipodes Island required a different approach. Most penguin colonies are located on the island foreshore, beneath steep cliffs rising vertically up to 150 m from sea level, with the exception of the south coast where the land slopes more gradually toward the island’s plateaus (Fig 1). This terrain rendered many colonies inaccessible to standard mission planning, so drone missions were launched from the cliff tops, with drones flying up to 120 meters below their launch sites. As the DroneDeploy mission planning system does not support negative flight altitudes, all surveys were conducted manually. Pilots flew drones in zig-zag patterns over breeding sites, capturing images at sufficient frequency to ensure at least 50% overlap, using topographic features or individual birds as reference points.

Additionally, some colonies on Antipodes Island are located partially or entirely in sea caves, making nadir surveys impossible. These were surveyed using a small, highly manoeuvrable FPV drone (DJI Avata 2, Shenzhen DJI Sciences and Technologies, Nanshan, Shenzhen, China), equipped with a 1/1.3-inch image sensor capable of recording 4K UHD video at 60 frames per second. Penguins inside caves were counted from still images extracted from multiple video fly-throughs.

### Generation of orthomosaics

Drone imagery was processed into high-resolution orthomosaics using photogrammetry, which identifies corresponding pixels in overlapping images and determines their spatial positions based on GPS data recorded by the drone, generating a three-dimensional point cloud. The images are then reprocessed to align with this point cloud, producing a single high-resolution, georeferenced composite image – an orthomosaic [28,29].

Orthomosaics were generated using the DroneDeploy cloud service (https://www.dronedeploy.com/), geographically projected in the WGS84/Pseudo-Mercator coordinate system (EPSG:3857), and downloaded in JPEG format with corresponding world files.

### Annotation of penguin colonies

Two annotation methods were used to count penguins in the orthomosaics. The 2022 Bounty Islands data were manually annotated, whereas for the 2024 Antipodes Islands surveys we used machine learning models integrated into the DroneDeploy service, trained on manually annotated orthomosaics from our 2022 Antipodes Islands expedition using the same annotation approach as for the Bounty Islands data described below. These models perform poorly in mixed penguin-albatross colonies, however, frequently misclassifying albatrosses as penguins and omitting individuals partially obscured by rocks or shadows – rendering them unsuitable for the Bounty Islands data, where manual annotation remained the more accurate approach.

### Bounty Island counts (2022 expedition)

Downloaded orthomosaics were analysed using the image annotation software DotDotGoose [version 1.6.0, https://github.com/persts/DotDotGoose; [30]]. Object classes were defined for each visible species, including Erect-crested penguins, Salvin’s albatrosses, Bounty Island shags [15], Fulmar prions, and fur seals. A grid overlay in DotDotGoose facilitated systematic annotation, with identified objects marked using colour-coded labels. Special care was taken to distinguish penguins from shags, using tail shape, crest feathers, and the presence of nesting material as distinguishing criteria.

After annotation, pixel coordinates were exported and converted into geographic coordinates using the orthomosaic world files. This transformation was automated using a Python command-line tool (https://github.com/persts/DotDotGoose/tree/main/extra_tools). To ensure reliability, three independent observers annotated each orthomosaic, generating three separate point datasets, which were then imported into ArcGIS Pro (version 3.0.3, Esri Inc., Redlands, California, USA).

Despite the high resolution of the orthomosaics, manual annotations inevitably contained some misclassifications – for example, shags mistaken for penguins, or shadows and rocks incorrectly labelled as birds. To enhance accuracy, a two-step verification process was applied. First, points marking the same species within a 15 cm radius and identified by all three observers were merged, using their centroid as the final object location. Second, points recorded by only one or two observers were manually reviewed and visually inspected to confirm or correct their classification, with incorrect points removed from the dataset.

To analyse penguin distribution, a kernel density analysis was conducted using the ‘adehabitatHR’ package [31] in R [32], with the 90th percentile contour used to delineate colony boundaries.

### Antipodes Islands counts (2024 expedition)

Orthomosaics were annotated using the ‘Annotation AI’ tool in DroneDeploy, a machine learning model developed using our manually annotated 2022 Antipodes Islands orthomosaics – produced using the same annotation approach as described above for the Bounty Islands. Leveraging DroneDeploy’s cloud infrastructure, the tool automatically identified and georeferenced individual penguins on processed orthomosaics. The resulting point data were downloaded in GeoJSON format (https://geojson.org/) and imported into ArcGIS Pro for validation. Each point was visually inspected, with misclassifications removed and missed penguins manually added.

Erect-crested and Eastern Rockhopper penguins occasionally breed in close proximity on the Antipodes Islands, but the DroneDeploy models did not distinguish between the two species. This differentiation was conducted manually during validation, based on crest shape and thickness – straight and thick in Erect-crested penguins, drooping and thin in Rockhoppers – as well as body size, Rockhoppers being approximately half the size of Erect-crested penguins. Rockhopper nests were also typically found on the fringes of Erect-crested colonies, providing an additional spatial cue for classification.

All identified penguins were grouped by colony, referencing descriptors established in a 2011 survey [27] to allow comparison with historical ground count data. As that report provided only rough geographic coordinates and basic maps, and colony boundaries had shifted naturally over the intervening decade, colonies were visually grouped to align with the most probable historical descriptors. Kernel density analysis was conducted following the same approach as for the Bounty Islands, with the 90th percentile contour used to delineate colony boundaries, providing reference points for future surveys.

For cave colonies, still images extracted from 4K video footage recorded by the FPV drone were manually counted using DotDotGoose as described above. Precise spatial data could not be recorded due to the absence of GPS reception inside caves; penguin locations were therefore estimated relative to one another and corresponding point data created manually in ArcGIS Pro. As a result, kernel density boundaries for cave colonies represent approximate distributions rather than precise spatial measurements.

### Ground-truthing counts and estimation of nest numbers

Drone imagery alone does not allow identification of active nests, as Erect-crested penguins use little nesting material and nest contents remain largely invisible from above, particularly early in the breeding season when adults are incubating eggs or brooding small chicks. Ground-truthing counts of penguin nests were therefore conducted to estimate breeding population size at both island groups.

On the Bounty Islands, all penguin nests on Proclamation Island were counted by two observers using tally counters over two days. Each counted nest was marked with a dab of blue paint to prevent double-counting. This was possible as penguins were still incubating eggs and remained on their nests when approached by observers.

The ratio of ground-counted nests to drone-counted birds was then used to estimate the breeding population across the entire archipelago, assuming the ratio determined for Proclamation Island was representative of the archipelago as a whole at that stage of the breeding cycle. While nest density varies among Bounty Islands [14], population trends are not expected to differ substantially between islands, since all are exposed to similar broad-scale drivers (oceanographic conditions, similar co-breeding seabird assemblages).

On the Antipodes Islands, the situation was more complex. The more than 80 Erect-crested penguin colonies distributed around the coastline required all sites to be visited on foot for drone surveys. Combined with frequently adverse weather conditions, particularly high winds and fog, surveying all colonies took considerably longer: the first mission was flown on 16 October 2024 and the final on 4 December 2024, spanning 50 days. Over this period, penguins progressed from egg incubation to the chick-guard stage, with hatching beginning in the second week of November, resulting in an increasing number of adults present in colonies. To account for these seasonal changes, ground counts coinciding with drone surveys were conducted throughout the season to obtain nest-to-penguin ratios representative of each breeding stage.

Full ground counts of entire colonies were not feasible due to time constraints, as survey teams needed to walk to and from colonies within a single day. Instead, subsections of colonies were surveyed from elevated vantage points outside the colony boundary. Two observers independently counted nests – identified by penguins in incubation posture or by visible eggs or chicks – and the total number of adult penguins present, using tally counters. Counts were accepted when the two observers differed by no more than two nests. The nest-to-penguin ratios obtained across the season were modelled as a linear function of survey date (expressed as day-of-year), and the resulting function was used to derive date-specific correction factors applied to penguin counts from each orthomosaic to estimate nest numbers.

Nest densities for both island groups were calculated as the number of nests relative to the area of the corresponding colony’s kernel density polygon.

### Estimating population trends

To assess long-term population trajectories of Erect-crested penguins on the Bounty and Antipodes Islands, historical nest count data were collated from published sources and grey literature. Despite being the least visited of New Zealand’s subantarctic island groups, the Bounty Islands have comparatively robust and methodologically consistent data. Full ground counts of nests on Proclamation Island were conducted in 1997, 2004, 2011, and 2019 using the same ground-counting methodology as employed in our 2022 survey, providing a small but reliable time series. These data were used as a proxy for the trajectory of the wider Bounty Islands population. A published estimate from 1978 [12] was considered for context but excluded from formal trend analysis.

Historical data from the Antipodes Islands are sparser and less consistent, ranging from rough estimates [13] to partial or binocular counts [10,27,33]; all rely to varying degrees on extrapolation from partial counts or indirect methods and therefore carry substantial methodological uncertainty. A partial survey conducted in 2014 [34] covered only a subset of colonies and did not provide a full-archipelago estimate; it was therefore excluded from trend analysis. Given the limitations of the historical data, the resulting regression should be interpreted as indicative of the direction and approximate magnitude of population change rather than a precise rate of decline.

For both archipelagos, population trends were assessed by fitting log-linear regression models of nest counts against year:


$$\begin{array}{c} ln(N_{t})=\alpha +\beta t+\varepsilon_{t} \end{array}$$

where *N*_*t*_ is the number of nests in year *t*, *α* is the intercept, and *ε*_*t*_ is the error term. The slope parameter *β* describes the average change in log nest counts per year, from which the average proportional annual change (APC) was calculated as:


$$\begin{array}{c} \text{APC }=e^{\beta }-\text{1}. \end{array}$$

Expressed as a percentage:


$$\begin{array}{c} \text{APC }(\%{\text{yr}}^{-\text{1}})=(e^{\beta }-\text{1})\times \text{ 100}. \end{array}$$

Statistical analysis was carried out in R v.4.2.2.

## Results

### Bounty Islands

Sixteen drone missions were conducted on the Bounty Islands between 10 and 15 November 2022 (Table 1). Seventeen of the 18 major islands, islets, and rock stacks of the archipelago were surveyed; only Con Island was not covered due to logistical constraints and the fact that the island is completely awash in high seas. Missions were flown around midday (mean = 13:27 h, range: 08:58–16:53 h), when the sun was close to the zenith to minimise shadows from animals and topography that could complicate image analysis. Flight duration averaged 22 min (range: 8−53 min, n = 16), and each mission produced on average 430 images (range: 188−1,026), yielding 5,610 images in total for orthomosaic generation and analysis.

Penguins were recorded on 12 islands across which a total of 32,199 penguins were counted (Table 1). Most (89.8%, n = 28,923) birds were found on the islands of the Main Group, with smaller proportions on the Centre Group (8.6%, n = 2,768) and Eastern Group (1.6%, n = 508) (Fig 3). Depot Island alone had over half of all penguins (54.4%, n = 17,502). Colony outlines and orthomosaics are accessible via https://doi.org/10.5281/zenodo.19562251.

**Fig 3:**
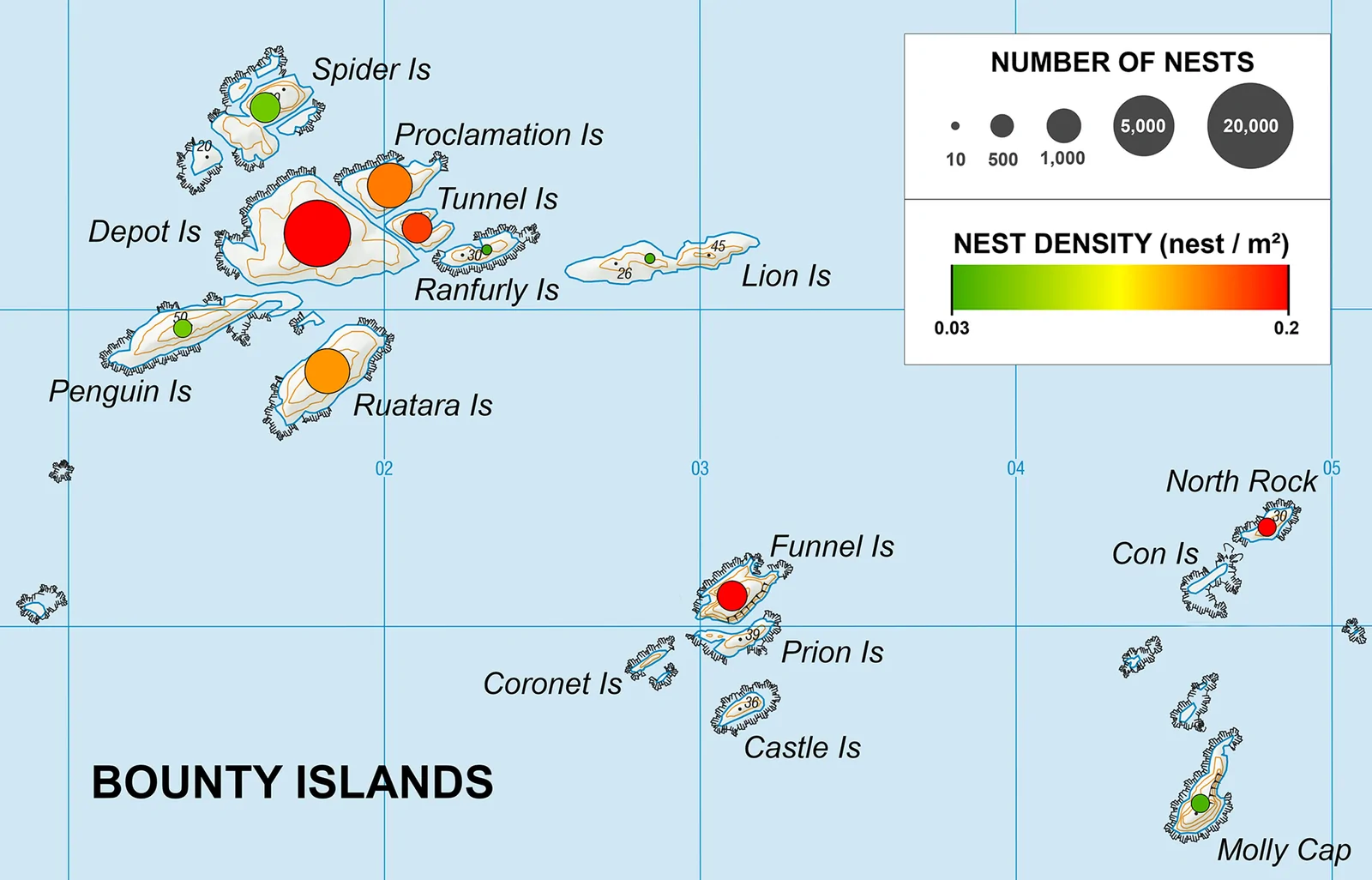
Estimated nest numbers and densities of Erect-crested penguins on the Bounty Islands in 2022. Estimates were derived from drone surveys and ground truthing counts conducted on Proclamation Island between 10 and 15 November 2022. Bubble symbols are sized proportionally to estimated nest numbers, colour indicates nest density (Table 1). Basemap: LINZ Topographic map (© Land Information New Zealand, CC BY 4.0).

Ground counts of penguin nests were carried out on Proclamation Island on 11 and 14 November 2022, yielding *N*_*g*_ = 2,776 nests, alongside a simultaneous drone-based count of *P*_*d,ref*_ = 3,216 penguins on the same island. As the entire archipelago was surveyed within a narrow window during incubation, when the nest-to-penguin ratio remains effectively constant, a single correction factor was calculated as:


$$\begin{array}{c} =N_{g}/P_{d,ref}=\text{ 2776 }/\text{ 3216 }=\text{0}.\text{863} \end{array}$$

This factor was applied uniformly to the drone-based penguin count *P*_*d,i*_ obtained for each island *i* across the archipelago to derive the estimated nest number for that island:


$$\begin{array}{c} N_{i}=r\times P_{d,i} \end{array}$$

Applied across all surveyed islands, this yielded a total estimate of 27,788 nests.

Breeding distribution across the archipelago covered 180,311 m^2^, corresponding to 40.2% of the total available landmass (S1 Table). Mean nest densities varied from very low on Ranfurly Island (0.03 nests/m^2^) to 0.20 nests/m^2^ on several islands (Depot, Funnel, North Rock) with an overall nest density of 0.15 nests/m^2^ (Table 1, Fig 3).

### Antipodes Islands

Between 16 October and 4 December 2024, 29 drone missions were flown across the Antipodes Islands, covering all known breeding colonies of Erect-crested penguins (Table 2). Missions were generally flown in the early afternoon (mean: 13:43 h, range: 10:57–18:21 h). On average, each mission produced 340 images (range: 11−1,714), totalling 9,155 images. Apart from the mission covering the extensive southwest coast colony, which exceeded two hours, flight times ranged from 3 to 58 min (mean: 21 min).

**Table 2:** Summary of drone counts of Erect-crested penguins on the Antipodes Islands, New Zealand, October-December 2024. Penguin numbers were determined by machine learning model annotation by the DroneDeploy cloud service (see methods) with subsequent manual validation of orthomosaics generated from drone surveys. Nest numbers were estimated by applying a linear correction function derived from multiple ground counts; note that survey date is the determining factor of the linear correction function (see results). Penguin colony outlines were quantified using the 90% kernel density contour, which was also used to calculate breeding area size and nest density; nest densities were calculated in all cases as estimated nests divided by breeding area. Most colonies appear fragmented forming individual clusters of nests varying greatly in size; this fact is represented by the number of nest clusters forming each colony as well as their minimum and maximum size. Note that for Hannah’s Cave, the spatial extent could not be determined accurately due to the absence of GPS coverage inside the cave and was therefore excluded from the area calculation; approximate nest density for this colony was estimated from video footage by visual comparison with Orde Lees.

		Colony clusters						
Colony	Area (m²)	n	Min size (m²)	Max size (m²)	Penguins (n)	Estimated nests (n)	Nest density (nests/m²)	Survey date
Southwest Coast	14,391	47	2	8,086	16,859	12,627	0.88	06 Nov
Orde Lees	5,203	6	16	2,498	7,700	6,868	1.32	24 Oct
Stack Bay	2,004	20	5	108	2,388	1,684	0.84	10 Nov
South Bay	1,829	13	4	1,019	2,006	1,502	0.82	06 Nov
Mirounga Bay	869	8	16	217	1,147	999	1.15	26 Oct
Archway Island	1,126	2	11	1,115	1,012	814	0.72	01 Nov
Cathedral Bay	922	7	18	300	1,117	738	0.80	14 Nov
Mirounga Bay North	763	9	10	250	781	679	0.89	26 Oct
Anchorage Bay East	825	10	4	508	853	676	0.82	02 Nov
Reef Point	564	1	–	–	773	673	1.19	26 Oct
Windward Bay West	552	5	21	320	626	510	0.92	31 Oct
Anchorage Bay West	482	10	12	149	470	373	0.77	02 Nov
Orde Lees West	300	4	42	159	347	283	0.94	31 Oct
Alert Bay	341	11	6	53	282	236	0.69	29 Oct
Hannah’s Cave	(133)	1	–	–	256	200	~1.30	03 Nov
East Windward Island	207	1	–	–	306	182	0.88	20 Nov
Crater Bay	378	4	34	73	217	179	0.47	30 Oct
Ringdove Bay	179	5	10	66	226	174	0.97	04 Nov
Castle Cove	281	5	4	137	199	138	0.49	11 Nov
Stella Bay	123	1	–	–	126	110	0.89	26 Oct
Albatross Point	74	4	–	–	86	66	0.89	04 Nov
South Bay East	75	1	–	–	127	56	0.75	04 Dec
Bollons Island	73	2	–	–	45	36	0.49	01 Nov
Windward Bay East	62	1	–	–	18	15	0.24	31 Oct
Northwest Bay	11	1	–	–	11	9	0.82	03 Nov
Lee Shore	11	1	–	–	8	5	0.45	13 Nov
**Total**	**31,644**	**180**	**2**	**8,086**	**37,987**	**29,832**	**0.94**	**–**

Five cave colonies were surveyed using the FPV drone: Hannah’s Cave, South Bay Cave, the Dungeon within Castle Cove, and two small caves within Alert Bay. With the exception of Hannah’s Cave, which is entirely cave-based, all other cave-associated colonies include nests both inside and outside cave entrances, and are reported as combined colony totals. Across all five locations, 461 penguins were counted strictly inside caves, representing approximately 1.2% of the total Antipodes penguin count.

Breeding penguins were distributed in 26 colonies comprising 180 distinct clusters of nests. Colonies occurred around the main Antipodes Island, with additional colonies on Archway, Bollons, and East Windward Island (Fig 4). A total of 37,987 penguins were counted across all colonies. Nearly two-thirds (64.7%, n = 24,559) were concentrated in the extensive Southwest Coast and Orde Lees colonies; the remaining 24 colonies were much smaller, each contributing 0.02–6.29% (range: 8−2,388 penguins) of the total.

**Fig 4:**
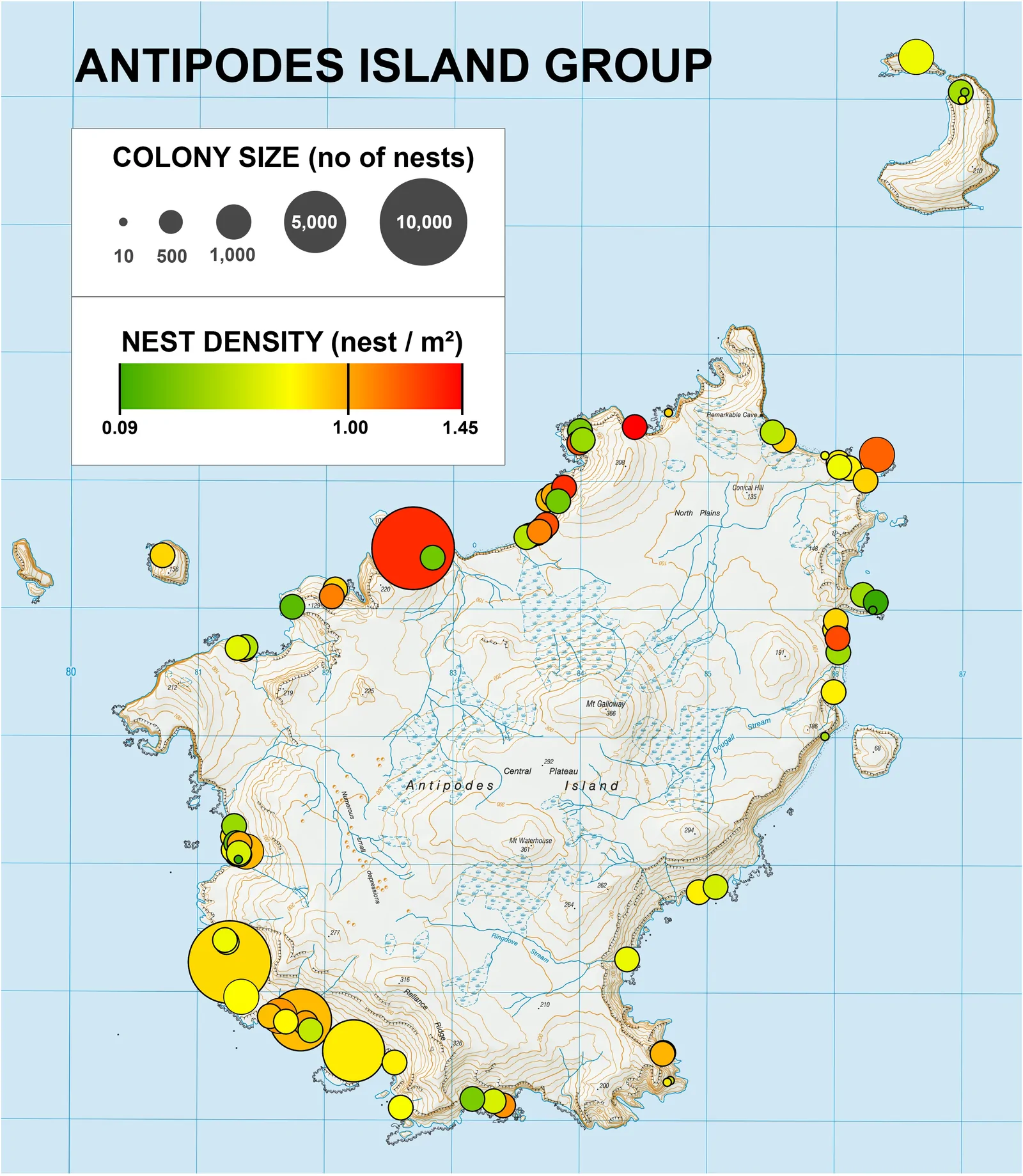
Colony distribution, size and nest densities of Erect-crested penguins on the Antipodes Island Group in 2024. Geographic location of major penguin colony clusters (n = 73) matched to survey sites described by Hiscock [27] are indicated by bubble symbols whose size is proportional to estimated nest numbers. Note that spatially proximate clusters are grouped into 26 named colonies in the text and Table 2. Bubble colour indicates nest density derived from 2024 drone surveys; clusters below the 1.0 nests/m^2^ threshold show more rapid decline, while those above show near-stable or slower-declining trajectories since 1995. Basemap: LINZ Topographic map (© Land Information New Zealand, CC BY 4.0).

Because the Antipodes Islands survey spanned 50 days (16 October – 4 December 2024) during which birds progressed from incubation through to chick-guard, the nest-to-penguin ratio could not be treated as constant. Instead, five paired ground counts of nests (*N*_*g*_) and penguins (*P*_*g*_) were conducted across the season (S2 Table), allowing the ratio *N*_*g*_/ *P*_*g*_ to be modelled as a function of survey date (day-of-year, *t*):


$$\begin{array}{c} r(t)=-0.011t+4.17 \end{array}$$

This date-specific ratio was then applied to the drone-based penguin count *P*_*d,i*_ obtained for each colony *i*, using that colony’s own survey date *t*_*i*_, to derive an estimated nest count:


$$\begin{array}{c} N_{i}=r(t_{i})\times P_{d,i} \end{array}$$

Summed across all colonies, this yielded a total estimate of 29,832 nests.

Despite the Antipodes archipelago being roughly 40 times larger than the Bounty Islands, Erect-crested penguins there breed in a far more spatially constrained footprint – 31,644 m^2^ compared to 180,311 m^2^ on the Bounties. This confinement to a fraction of the available coastline is reflected in markedly higher nest densities of 0.94 nests/m^2^ (range: 0.09–1.45 nests/m^2^; Fig 4) compared to 0.15 nests/m^2^ on the Bounty Islands.

### Population trajectories

On the Bounty Islands, a log-linear regression fitted to nest counts from Proclamation Island between 1997 and 2022 (Table 3) indicated no detectable trend in the population over the past 25 years (APC = −0.09% yr ^−1^, 95% CI: −0.55 to +0.38%, R^2^ ≈ 0.041). Full-archipelago nest estimates for the Antipodes Islands from 1995, 1998, 2011, and 2024, revealed a strong long-term population decline, with an average proportional annual decline of 1.50% yr^-^¹ (95% CI: −3.89 to +0.95%, R^2^ ≈ 0.779; Fig 5).

**Table 3:** Long-term trajectories of Erect-crested penguin colonies on Proclamation Island, Bounty Islands. Nest counts were all conducted using the same methodology employed during this study.

Year	Nest numbers	Data source
1997	2,774	Clarke et al. (1998) [35]
2004	2,788	De Roy & Amey (2004) [36]
2011	2,514	Mattern et al. (2021) [14]
2019	2,676	Mattern et al. (2021) [14]
2022	2,776	This study

**Fig 5:**
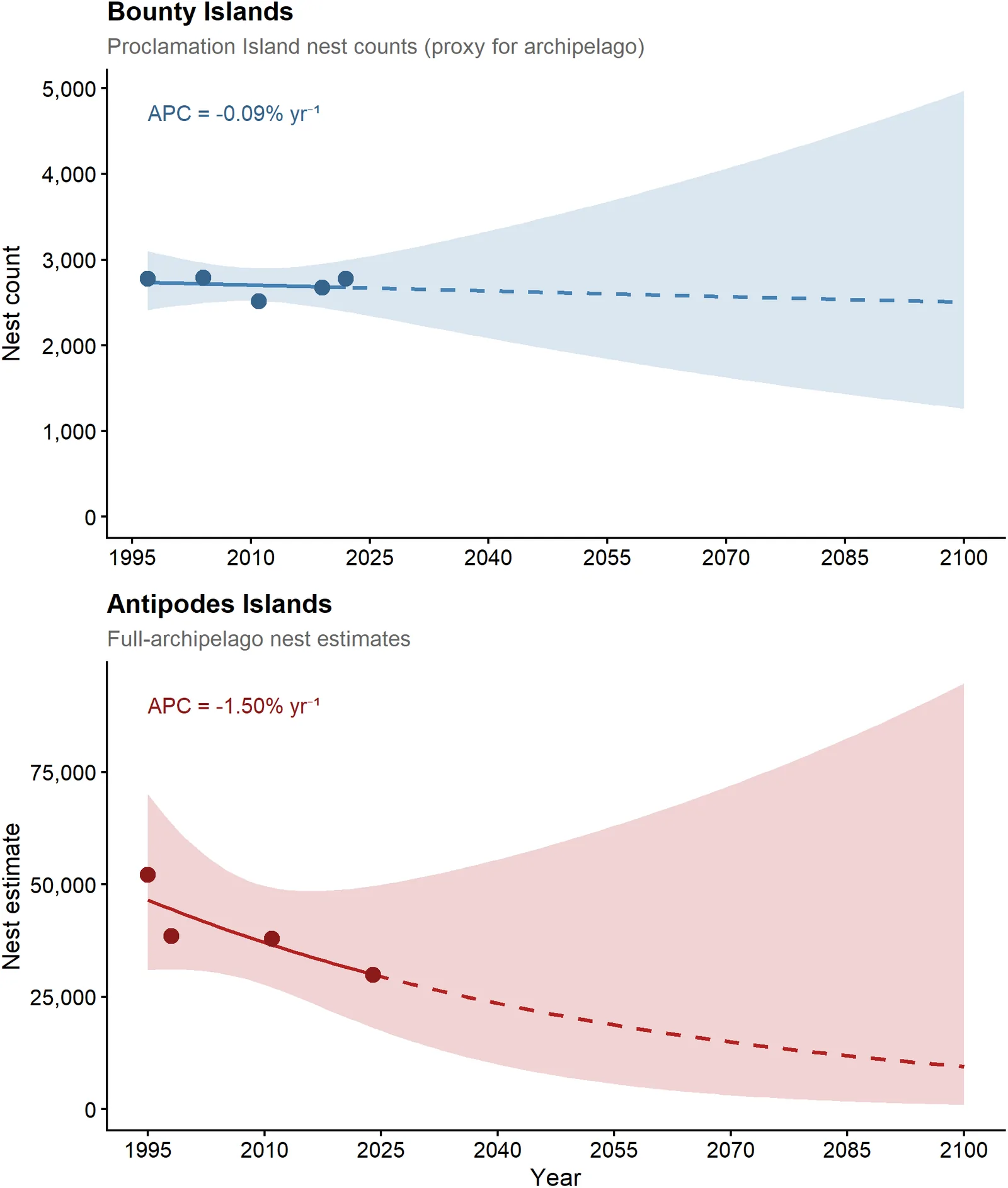
Long-term trajectories of Erect-crested penguin colonies on the Bounty and Antipodes Islands. Top: Ground nest counts on Proclamation Island (1997-2022), used as a proxy for the Bounty Islands archipelago. Bottom: Total nest estimates for the Antipodes Islands (1995-2024) derived from ground counts and drone surveys (see Methods). The 2014 Antipodes survey was omitted because it covered only a subset of colonies and did not provide a full-archipelago estimate. Log-linear regressions (solid lines) with 95% confidence intervals (shaded) were fitted to all available survey years and projected forward to 2100 to illustrate potential population trajectories.

Historical nest estimates were available for five Antipodes colonies dating back to 1995 (Table 4), enabling calculation of APC for each colony. A consistent pattern emerged: colonies with nest densities <1.0 nests/m^2^ showed markedly steeper declines than those above this threshold. Nearly 80% of all colonies fell below this threshold and are therefore likely experiencing higher rates of decline. Only Orde Lees, Reef Point, Hannah’s Cave, and Mirounga Bay feature nest densities above 1.0 nests/m^2^, suggesting approximately stable or less severe rates of decline (Fig 5).

**Table 4:** Long-term trajectories of Erect-crested penguin colonies on the Antipodes Islands. Nest estimates are shown for each survey year with a log-linear regression fitted to all available years to estimate the average annual proportional change (% yr ^−1^). Negative values indicate decline, positive values growth. Nest densities provided for the different sites were derived from 2024 drone surveys (this study); nest density for the entire Antipodes Island group is provided as median of all colonies. Data sources are provided below table.

Site	1995^1^	1998^2^	2011^3^	2014^4^	2024	Annual change (%)	Nest density 2024 (nests/m²)
Orde Lees	7,319	–	7,832	–	6,868	−0.19	1.32
Reef Point	713	654	578	505	673	−0.43	1.19
South Bay	2,376	–	1,920	–	1,502	−1.56	0.82
Anchorage Bay	2,779	–	2,048	1,673	1,049	−3.22	0.77
Stella Bay	330	303	251	222	110	−3.31	0.89
Antipodes Island group	52,081	38,540	37,900^5^	–	29,832	−1.50	0.84

^1^Tennyson & Taylor (2001), ^2^Davis et al. (2022), ^3^Hiscock (2013), ^4^Chilvers & Hiscock (2019), ^5^Wilson & Mattern (2019)

## Discussion

Drone-based surveys of the entire global range of the Erect-crested penguin reveal that the species’ two subantarctic breeding strongholds support broadly comparable numbers of breeding pairs – approximately 27,800 on the Bounty Islands and 29,800 on the Antipodes Islands. This stands in stark contrast to the 1978 estimates of 115,000 breeding pairs per island group [12,13] that form the foundation of the species’ current IUCN “Endangered” classification [24], and overturns the long-standing assumption that the Antipodes Islands represent the species’ dominant stronghold [11,18]. Combining these contemporary baselines with historical nest count data reveals a stark demographic divergence: whereas the Bounty Islands population has remained stable over at least the past 25 years, the Antipodes Islands population has declined by more than 40% since the mid-1990s. These findings inevitably necessitate a reassessment of the species’ global threat status – yet doing so exposes a deeper conundrum. Treating the two populations independently yields divergent threat classifications, while any improvement in the global ranking risks being misconstrued as vindication by decision makers for past inactivity and used as justification to relax existing fisheries restrictions – a prospect of particular concern given that the Bounty Islands, now potentially the species’ last stable stronghold, lie within one of the region’s most intensively fished areas.

### Divergent population trends

Historically, the Antipodes Islands were considered the more important of the two strongholds, with the estimated breeding population of 52,081 pairs in 1995 [37] being nearly twice the 27,956 pairs estimated for the Bounty Islands in 1997 [18,35]. Since then, two divergent population trajectories have become apparent. On the Bounty Islands, nest counts have remained essentially unchanged over 25 years, with a log-linear regression indicating no detectable trend (Fig 5). The reliability of this time series rests on the methodological consistency of Jacinda Amey’s ground counts [14,35,36], which established and followed robust survey protocols – a consistency independently confirmed by the close agreement between her 1997 archipelago-wide estimate of 27,956 pairs and our 2022 drone-derived estimate of 27,788 pairs, a difference of less than 1%. On the Antipodes Islands, by contrast, each successive survey has employed different methods and levels of coverage, rendering direct comparisons uncertain [8]. Nevertheless, the available data indicate a decline of approximately 43% between 1995 and 2024, a trajectory supported by log-linear regression across all available survey years (Fig 5).

The divergent trajectories of the two population strongholds inevitably raise the question of underlying mechanisms. Given that variability in penguin populations is generally associated with changes in oceanic habitat [e.g., 38–40], environmental differences between the two island groups are a natural starting point – despite their relative proximity.

### Potential mechanisms behind contrasting population trends

#### Differences in oceanic habitat.

Although separated by only ~200 km of open water, the Bounty and Antipodes Islands sit within oceanographically distinct environments. The Bounty Islands lie adjacent to the Bounty Trough to the north, where nutrient flux originating from the New Zealand mainland drives elevated phytoplankton productivity [41] and supports intensive commercial fishing activity [42; Global Fishing Watch, https://www.globalfishingwatch.org]. In contrast, chlorophyll concentrations around the Antipodes Islands indicate comparatively lower productivity typical for the New Zealand subantarctic region [43], suggesting potentially less favourable foraging conditions in the immediate vicinity of the colony. During the chick-rearing stage, however, foraging ranges and dive depths of birds from both island groups appear broadly comparable [44], suggesting that differences in local productivity do not translate straightforwardly into differential foraging conditions during the breeding season, and that food availability is unlikely to be a primary driver of the observed population divergence – at least during chick-rearing, when the quantities of krill transferred to chicks indicate resources are not limiting at either site.

Differences in oceanic habitat may, however, exert stronger demographic effects outside the breeding season. The difference of two to three weeks in breeding onset between the two archipelagos could indicate that the penguins’ annual cycles are determined by differing environmental conditions. Indeed, pre-moult satellite tracking of Erect-crested penguins from both island groups reveals a striking divergence in spatial behaviour (Mattern et al, unpublished data; interactive tracking map available at https://ptx.lat/ers24). Antipodes Island birds moved pronouncedly southward after breeding, reaching latitudes of up to 61.2°S travelling up to 1,287 km from the colony, with two individuals penetrating beyond 60°S into the Antarctic Polar Frontal Zone. In marked contrast, Bounty Islands birds remained in waters immediately surrounding the archipelago, with a mean maximum southward extent of only 48.6°S and a mean maximum distance of just 306 km from the colony. These pre-moult movements are likely a precursor to full winter migrations, and the divergence in spatial behaviour between the two populations is consistent with the broader pattern documented across New Zealand crested penguins [45–47].

Post-moult tracking of Erect-crested penguins and Eastern Rockhopper penguins from Antipodes Island using GLS loggers confirms an extended eastward winter migration into the Antarctic Polar Frontal Zone, with round trips averaging ~10,500 km over approximately 170 days [46]. By contrast, Snares penguins (*Eudyptes robustus*) and tawaki/Fiordland penguins (*Eudyptes pachyrhynchus*) – the closest relatives to Erect-crested penguins [48] – both migrate westward. Snares penguins remain largely north of the Subtropical Front in waters of comparatively stable productivity [46]; tawaki exhibit more flexible migration behaviour than other crested penguins, crossing several frontal zones during their principally westward migration – a pattern likely reflecting the species’ subantarctic origins despite its current temperate breeding range [47]. Whether Bounty Islands Erect-crested penguins might follow a similar westward strategy during winter remains unknown, but the concentrated pre-moult movements suggest their winter dispersal may also differ fundamentally from that of their Antipodes counterparts.

The Bounty Islands lie immediately south of the Subtropical Front, placing this productive frontal zone within easy reach of birds departing the archipelago – making a westward migration tracking the Subtropical Front, analogous to that of Snares penguins, a plausible alternative to the eastward dispersal within the polar frontal zone documented in Antipodes Island birds. If so, differential exposure to inter-annual variability within the Antarctic Polar Frontal Zone – increasingly disrupted by marine heatwaves across the South Pacific [e.g., 49] – could translate into lower survival rates in Antipodes Island birds during the non-breeding season, contributing to the divergent population trajectories observed between the two island groups. Resolving this question through dedicated simultaneous winter tracking of Bounty and Antipodes Islands birds is a clear priority.

Beyond effects on adult survival, differences in migratory behaviour between the two populations could also affect recruitment if juveniles disperse into similar oceanic zones as adults. Although fledgling dispersal in seabirds has generally been found to be independent of adult movements [e.g., 50–53], crested penguins remain unstudied in this regard. If juveniles from the Antipodes Islands tend to disperse into the polar frontal zone similar to adults – whether through inherited predisposition [e.g., 54, 55] or environmental forcing [e.g., 56] – they would have to cope with variable oceanic productivity of that region during their critical first years at sea, potentially suppressing recruitment rates. This would likely compound reduced breeding output resulting from predation pressure on land, creating a dual demographic burden that could accelerate the decline documented on the Antipodes Islands.

### Skua predation, colony fragmentation, and the Allee effect

A key asymmetry between the two archipelagos is the presence of a resident breeding population of Subantarctic skua on the Antipodes Islands, estimated at up to 100 pairs [13], with nest sites typically positioned directly above or adjacent to penguin colonies. No equivalent resident skua population exists on the Bounty Islands, where the vegetation-free terrain and dense mixed assemblages of penguins, albatrosses, and fur seals appear to offer no suitable breeding conditions for skuas [cf. 57]. There is no record of a breeding population of skua at the archipelago and any vagrant individuals that do visit are unlikely to exert sustained predation pressure comparable to that of resident breeders.

The demographic consequences of skua predation on the Antipodes Islands are particularly apparent during the transition from the chick guard to crèching stages of breeding. Following hatching, male penguins guard chicks continuously while females make short provisioning trips of one to two days [8]. Around three weeks post-hatching, most males depart to forage for the first time in over a month – a departure that is broadly synchronised across colonies. At this transition from the guard to post-guard stage, chicks are suddenly left unattended for extended periods, forcing them to form crèches. This transitional period represents maximum vulnerability: chicks are still small enough to be carried off in flight by skuas, and the abrupt reduction in attending adults means that coordinated attacks by multiple skuas can overwhelm whatever defensive capacity remains in the colony. Carcasses of predated chicks are a conspicuous feature of colony margins and adjacent beaches on the Antipodes Islands toward the end of the breeding season, whereas dead chicks are far less commonly observed on the Bounty Islands, where skua pressure is absent.

The severity of skua predation appears to be dependent on colony size and nest density [44], generating a classic Allee effect: as colony density falls, collective defence weakens, and predation pressure per nest increases [58,59]. In densely packed colonies such as Orde Lees (1.32 nests/m^2^, Table 4), skuas seem to be confined to the colony periphery, leaving the majority of nests beyond reach of aerial attack. As nest density decreases, however, landing opportunities within the colony increase, exposing even centrally positioned chicks to predation. This is reflected in the colony-level trend data: colonies with nest densities >1.0 nests/m^2^ show near-stable or only modestly declining trajectories, while those below this threshold decline markedly more steeply (Table 4, Fig 4). Approximately 80% of Antipodes colonies currently fall below this density threshold, suggesting that the majority of the population is exposed to elevated predation pressure.

The predation pressure on Erect-crested penguins has likely intensified over recent decades as a consequence of dramatic changes in the prey base available to Antipodes skuas. Early accounts from the 1950 Alert Expedition, described in detail by Taylor [13], record Eastern Rockhopper penguins as “far outnumbering Erect-crested penguins.” For local skuas, this superabundance of Rockhopper prey likely provided the resource base that supported specialisation on penguins as a primary food source. However, the latter half of the twentieth century saw a catastrophic collapse of Rockhopper penguin populations across the New Zealand region [38,60], a trajectory mirrored on the Antipodes Islands [13]. The most recent estimate places the Antipodes Rockhopper population at <1,000 breeding pairs [23] – a remnant of the vast assemblages described in the 1950s. As Rockhopper numbers dwindled, Erect-crested penguins likely became an increasingly important target for resident skuas. Since skuas rarely kill healthy adult penguins, the predation pressure falls predominantly on eggs and chicks [61] adding a substantial suppressing factor to breeding success and subsequent recruitment.

The interaction between skua predation and declining colony density creates a self-reinforcing dynamic. As penguin numbers fall, formerly continuous colonies fragment into smaller, spatially discrete clusters separated by encroaching vegetation that recolonises ground vacated by penguins (Fig 6). Smaller, more isolated clusters offer fewer guarding adults per unit area, enhancing skua access and reducing the effectiveness of collective defence – driving further reproductive failure and accelerating fragmentation. In the most extreme cases, small clusters of fewer than approximately ten breeding pairs can experience near-complete chick mortality, as skuas methodically work through the crèche before adult return rates can provide meaningful protection.

**Fig 6:**
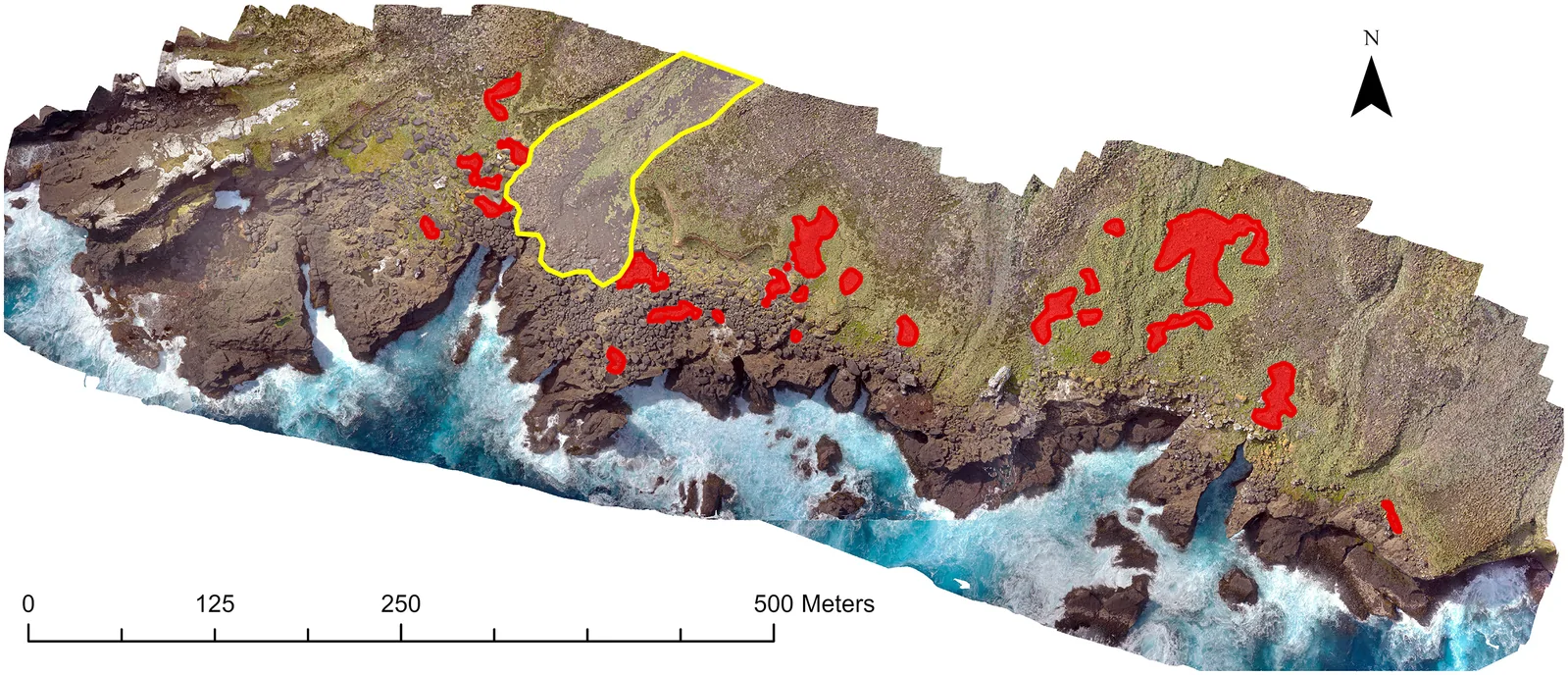
Example of fragmentation of Erect-crested penguin colonies on the south coast of Antipodes Island in 2024. Orthomosaic of the eastern region of the Southwest coast comprising an estimated 2,378 nests distributed across 25 clusters with nest numbers ranging from 19 to 1,309 nests. A landslide that buried a substantial number of active nests during the 2014 breeding season [34] is indicated by a yellow polygon.

### Topographic factors

Landslides and rockfalls contribute to colony fragmentation, but their role as a driver of population change [34] should not be overstated. A substantial rockfall during the winter of 2024 buried an estimated 50 pairs’ worth of nesting habitat at Mirounga Bay, yet penguins established nests directly on the debris in the following season with no significant decline in numbers [23] demonstrating resilience to physical disruption when it occurs outside the breeding season.

Direct mortality becomes a factor when habitat disruption coincides with active breeding. Chilvers and Hiscock [34] documented the consequences of a severe storm in January 2014 – the most extreme weather event recorded at the Antipodes Islands in at least 20 years – that triggered landslides affecting 44% of surveyed penguin colonies. Colonies sustaining area losses exceeding 75% saw breeding pair numbers decline by approximately 40% in the following season, compared with around 12% in unaffected colonies. For such events to be relevant at a population level, though, two conditions must align: exceptional storm severity sufficient to trigger widespread landslides, and timing within the narrow window when large numbers of adult penguins are ashore. The 2014 event met both conditions but remains without precedent in the decades either before or since. As a driver of long-term decline, direct landslide mortality is therefore likely of minor importance relative to the chronic processes of predation and density-dependent fragmentation.

The lasting consequences of landslides, however, extend beyond direct mortality. By altering colony topography – fragmenting contiguous habitat into isolated clusters and opening gaps that improve skua access (Fig 6) – episodic disturbance can push colonies already near critical density thresholds further below them, accelerating the Allee-driven decline. Storm events in the Southern Ocean are projected to increase in frequency and severity [62], raising the prospect of more frequent topographic disruption compounding an already self-reinforcing dynamic.

The mechanisms driving the divergent population trajectories are not fully resolved, but the interplay of oceanographic differences, differential predation pressure, and episodic habitat disturbance is clearly tilting the two populations in opposite directions. Given the species’ binary population distribution, this creates a direct challenge for its threat classification which at the moment is based on historical records determined with varying methodologies and accuracy [18].

### Validity of historical records

Although it has been suggested that Erect-crested penguins might have been more widely distributed in the NZ subantarctic region [11] a more recent review highlights that breeding outside of the Antipodes and Bounty Islands was quasi insignificant [8]. In the 1940s and 1950s, a small number of pairs bred in amongst Eastern Rockhopper penguins on Campbell Island [63], one pair was reported breeding on the New Zealand mainland [64] and a few pairs have been noted in the past on Disappointment Island in the Auckland Island group [8]. As such, the Antipodes and Bounty Island groups represent the species’ core breeding distribution.

The Erect-crested penguin’s current “Endangered” classification rests principally on the assumption that both island populations declined by more than 50% between 1978 and the mid-1990s. This assumption largely hinges on the validity of the 1978 population estimates and requires careful consideration.

### Bounty Islands

For the Bounty Islands, Robertson & van Tets [12] estimated 115,000 breeding pairs based on penguin nest densities determined opportunistically in some sections of Proclamation Island and extrapolated over the total available landmass. To achieve an estimate of 115,000 breeding pairs within the current breeding footprint of 180,311 m^2^ would require a nest density of approximately 0.64 nests/m^2^ – four times the observed density of 0.15 nests/m^2^ and well above the maximum recorded on any individual island – rendering the 1978 figure implausible given the co-existence with other ground breeding species. All subsequent surveys of the Bounty Islands, however, ranging from the first comprehensive ground count in 1997 to this study, have consistently reported breeding populations of around 27,000 pairs. Comparison of aerial photographs taken when the 1978 expedition took place with drone orthomosaics of the same areas of Proclamation Island found comparable penguin nest numbers [14]. This suggests that the Bounty Islands population has been stable at around 27,000 pairs for at least 25 years, making it likely that the 1978 figure substantially overstated the actual population size.

### Antipodes Islands

The validity of the Antipodes Islands figure is even less clear. Taylor [13] provides only a “very rough population estimate” of 115,000 breeding pairs of Erect-crested penguins derived from his first full mapping of penguin colonies in 1978. No methodology is described, and examination of Taylor’s field notebooks, hand-drawn maps, and photo slides from his estate yields no insight into how this figure was derived. Notably, Taylor cautioned that the perceived decrease in Erect-crested penguin numbers may have been exaggerated by comparing his rough 1978 estimate with the more accurate counts that followed later. It is also conspicuous that the Antipodes figure is identical to the Robertson & van Tets [12] estimate for the Bounty Islands – a remarkable coincidence given that the two archipelagos differ in size, topography, and habitat. The Bounty Island expedition is extensively referenced in Taylor [13] which opens the possibility that the published Antipodes figure was erroneously carried over from the Bounty Islands results during the writing or editing of the book rather than representing the true estimate.

That real decline occurred on the Antipodes Islands in the second half of the 20th century is not in question. Photos published in Taylor [13] document that Erect-crested penguin colonies were substantially more extensive in the late 1960s than they are today. This is also supported by comparison of satellite imagery with drone orthomosaics from this study: the vegetation-free footprint of the Orde Lees colony shrank by 27% between 2005 and 2022, while Southwest Coast colonies contracted by 62% over the same period and fragmented into several discrete clusters [19; Fig 6].

In summary, both 1978 population estimates for the Erect-crested penguin must be considered inaccurate, and neither has the robustness required for a valid threat assessment. The implied decline, on which the current “Endangered” classification is based [24], is therefore principally an artefact of comparing unreliable historical figures with later, more rigorous counts rather than evidence of a genuine population collapse of that magnitude.

### The IUCN threat ranking conundrum

The Erect-crested penguin was most recently assessed as “Endangered” on the IUCN Red List in 2020, under criteria A2b; B1ab(ii,v)+2ab(ii,v) [24]. The population size criterion (A2) is triggered by an observed reduction exceeding 50% over three generations – a threshold met by inclusion of the unreliable 1978 estimates. The range criteria (B1, B2) reflect the species’ extreme geographic restriction to two small island groups, a biological reality that is unlikely to change.

Reassessing the species against the more reliable data available since the mid-1990s produces a markedly different picture. Using the 1997 Bounty Islands ground count of 27,956 pairs [18] and the 1995 Antipodes total of 52,081 pairs [37] as a combined baseline of approximately 80,000 breeding pairs, the current global population of around 57,600 pairs (this study) represents a reduction of roughly 28%. This falls just below the 30% threshold for “Vulnerable” under Criterion A; together with the species' restricted range under Criterion B, a global listing of “Vulnerable” would therefore be likely – an improved threat ranking compared to its current listing.

However, this approach “averages” the divergent population developments across the species’ binary range effectively biasing the global assessment towards the stable Bounty Island population and, thus, masking the ongoing significant decline on the Antipodes Islands. Applying IUCN criteria independently to each population, however, results in contrasting threat assessments.

The Bounty Islands population – stable for at least 25 years and now established as broadly equal in size to its Antipodes counterpart – does not meet the threshold for any threatened category under Criterion A. It would, however, qualify as “Vulnerable” under Criterion B on account of its limited geographic extent: the entire penguin population is confined to a single 50-hectare archipelago, where any catastrophic event – a major oil spill, an extreme storm, or a disease outbreak – could eliminate a substantial proportion of the global population.

The Antipodes Islands population presents the opposite case. Its cumulative decline of approximately 43% since 1995 exceeds the 30% reduction required for “Vulnerable” status under Criterion A2. However, the confidence interval around the regression-derived APC is wide (95% CI: −3.89 to +0.95% yr ^−1^), reflecting the sparse and methodologically inconsistent nature of the historical survey data. At the lower bound of this interval, the cumulative decline over three generations would reach approximately 71%, well into the “Endangered” range. The IUCN guidelines explicitly recommend applying the precautionary principle where uncertainty spans a threshold: if the plausible range of decline rates includes values that would trigger a higher category, that category is justified [5]. Given that the uncertainty in the Antipodes trend estimate does exactly that, and given the well-documented ongoing declines in area of occupancy under Criterion B2, paired with potentially accelerated declines resulting from the Allee effect due to ongoing colony fragmentation, the Antipodes Islands population warrants “Endangered” classification – the very status currently applied to the species as a whole, but for the wrong reasons and at the wrong scale.

The divergent population trajectories, resulting in disparate threat rankings at the subpopulation level, raise both a scientific and a communicative conundrum. At the global level, aggregating two populations on opposing demographic trajectories into a single headline status accurately describes neither of them. A revised global listing of “Vulnerable”, however technically defensible, risks being interpreted as reassuring news. The species’ “Endangered” status forms part of the conservation case for maintaining and strengthening protections around the Bounty Islands [65] – protections that were already substantially compromised by commercial fishing interests during their establishment, with over 40% of the territorial sea excluded from protection to accommodate the existing ling longline fishery [66; Fig 1]. In a policy environment where economic priorities frequently outweigh conservation considerations [e.g., 67, 68] – a trend also evident in New Zealand [e.g., 69–72] – a global downlisting, however scientifically justified at the species level, risks being weaponised by commercial interests as justification for relaxing these already-limited protections, precisely at the moment when safeguarding the Bounty Islands population has become most critical. This prospect is of particular concern given that the Bounty Islands lie within one of the region’s most intensively fished areas [42]. The tension between conservation urgency at the population level and a threat categorisation at a global scale creates a communication and management paradox.

This is not the result of an oversight on the part of the IUCN guidelines – regional assessment provisions exist precisely to prevent globally aggregated statuses from masking local extinction risk [5]. However, these provisions are designed primarily for cases where a species is secure across most of its range but threatened in a defined subset of it. They offer less clear guidance for taxa such as the Erect-crested penguin, whose entire global population is confined to a small number of sites of comparable size following divergent trajectories. More fundamentally, the Red List’s value as a communication tool rests on its reduction of complex population data into a single category, from “Least Concern” to “Critically Endangered”, a simplicity that is also its principal weakness. The nuance needed to describe a species’ status in greater detail is not absent from the Red List process; it exists in the accompanying assessment justification and supporting documentation. In practice, however, this is the fine print: the category itself is what gets cited, reported, and acted upon by decision-makers, while the qualifying detail that reveals a more complicated reality goes largely unread. Cases of this kind are unlikely to be unique to the Erect-crested penguin; addressing this issue is unlikely to be achieved by expecting closer engagement with supporting documentation from an audience that, in practice, does not consult it – the responsibility for surfacing this complexity rests with the assessment and reporting system itself, not with those who rely on its output.

### Schrödinger’s Penguin – two states, one species

The Schrödinger’s Cat thought experiment derives its power from a simple paradox: two contradictory states coexist until observation forces a resolution. For the Erect-crested penguin, opening the proverbial box has not resolved the paradox – it has deepened it. The species exists simultaneously in two demographic states: stable on the Bounty Islands, declining on the Antipodes, and no single threat ranking can accurately describe both.

The Erect-crested penguin has carried an “Endangered” listing for 25 years [24]. During that time, it has attracted virtually no targeted conservation attention – no recovery plan, no dedicated management measures, no meaningful public profile [10,11]. For a species breeding exclusively on two remote, uninhabited island groups largely unknown beyond a small community of specialists, this is probably unsurprising.

This contrasts markedly with the other endemic penguin species in New Zealand, also ranked “Endangered” – the hoiho/yellow-eyed penguin (*Megadyptes antipodes*). The species breeds on the mainland South Island and subantarctic Auckland and Campbell Islands [73], with the mainland population projected to face regional extinction by 2070 under climate change scenarios [40] – a trajectory that may already be unfolding faster than modelled [74]. The status of the subantarctic island populations remains poorly understood [75], mirroring the data gaps that have long hampered assessment of the Erect-crested penguin. Yet the hoiho’s mainland presence has made it visible, its visibility has generated public concern [76], and that concern has sustained decades of intensive conservation effort by government agencies, non-governmental organisations, and community groups [77]. This underscores that an “Endangered” listing may be necessary but not sufficient – it requires a public that knows and cares about the species to translate into action.

For the Erect-crested penguin, the “Endangered” listing has not achieved this; a revised global listing of “Vulnerable” would instead weaken the basis for any precautionary management. The Antipodes Islands population is in sustained decline, with the processes driving that decline showing no sign of abating. Some of the larger, denser colonies such as Orde Lees and Reef Point may persist for the foreseeable future, but the archipelago as a species stronghold is diminishing. The Bounty Islands, by contrast, likely represent the species’ long-term demographic anchor. Protecting that population – from fisheries pressure and the erosion of the regulatory framework that currently limits it [71,72,78], and from the risk of catastrophic stochastic events – is the most immediate conservation priority.

Unlike Schrödinger’s cat, whose fate is determined the moment the box is opened, the Erect-crested penguin defies resolution – the two states are real, simultaneous, and demand management responses that no single threat ranking can capture.

## Supporting information

S1 Table Breeding occupancy of Erect-crested penguins on the Bounty Island archipelago, New Zealand, November 2022. Land area of the various islands was derived from digital elevation models (DEM) generated from orthomosaics. Breeding areas were determined as the size of the 90% kernel density contour of penguins on each island.
(DOCX)

S2 Table Ground counts to determine correction factor for breeding pair estimates of Erect-crested penguins on Antipodes Island, October-December 2024. All counts were conducted on subsections of colonies agreed upon by observers prior to counts. To calculate the correction factor, average ratios for each day of the year were calculated.
(DOCX)

S1 File Inclusivity in global research questionnaire. (DOCX)
