# Supplementary material for: Schrödinger’s Penguin? Divergent population trajectories and the challenge of assessing the threat status of the Erect-crested penguin
Source: PLoS One. 2026 Oct 21;21(10):e0359075. doi: 10.1371/journal.pone.0359075 (PMC13686514; doi:10.1371/journal.pone.0359075)
Supplement: Supplementary file 1 [file pone.0359075.s001.docx]

**S1 Table. Breeding occupancy of Erect-crested penguins on the Bounty Island archipelago, New Zealand, November 2022.** Land area of the various islands was derived from digital elevation models (DEM) generated from orthomosaics. Breeding areas were determined as the size of the 90% kernel density contour of penguins on each island.

| **Island** | **Land area (m²)** | **Breeding area (m²)** | **Coverage (%)** |
| --- | --- | --- | --- |
| Tunnel Is | 16,402 | 10,206 | 62.2 |
| Depot Is | 126,832 | 74,079 | 58.4 |
| Spider Is | 40,080 | 23,357 | 58.3 |
| Proclamation Is | 32,615 | 17,835 | 54.7 |
| Funnel Is | 27,159 | 12,033 | 44.3 |
| Ruatara Is | 58,039 | 18,588 | 32.0 |
| Molly Cap | 24,084 | 6,292 | 26.1 |
| Penguin Is | 51,017 | 12,806 | 25.1 |
| Ranfurly Is | 14,683 | 2,651 | 18.1 |
| North Rock | 8,357 | 1,097 | 13.1 |
| Lion Is | 48,965 | 1,367 | 2.8 |
| **TOTAL** | **448,233** | **180,311** | **40.2** |
