# Supplementary material for: Schrödinger’s Penguin? Divergent population trajectories and the challenge of assessing the threat status of the Erect-crested penguin
Source: PLoS One. 2026 Oct 21;21(10):e0359075. doi: 10.1371/journal.pone.0359075 (PMC13686514; doi:10.1371/journal.pone.0359075)
Supplement: Supplementary file 2 [file pone.0359075.s002.docx]

**S2 Table. Ground counts to determine correction factor for breeding pair estimates of Erect-crested penguins on Antipodes Island, October-December 2024.** All counts were conducted on subsections of colonies agreed upon by observers prior to counts. To calculate the correction factor, average ratios for each day of the year were calculated.

| **Date** | **Day of Year** | **Site** | **Birds on ground** | **Number of nests** | **Ratio** |
| --- | --- | --- | --- | --- | --- |
| 26/10/2024 | 300 | Mirounga Bay North (Top) | 28 | 24 | 0.85 |
| 26/10/2024 | 300 | Mirounga Bay North (West) | 19 | 17 | 0.90 |
| 29/10/2024 | 303 | Anchorage Bay | 80 | 64 | 0.80 |
| 06/11/2024 | 311 | Southwest Coast | 106 | 82 | 0.77 |
| 06/11/2024 | 311 | South Coast | 60 | 47 | 0.78 |
| 06/11/2024 | 311 | South Bay | 45 | 33 | 0.72 |
| 11/11/2024 | 315 | Stack Bay | 37 | 24 | 0.66 |
| 04/12/2024 | 338 | Anchorage Bay | 144 | 62 | 0.43 |
