# Supplementary material for: Schrödinger’s Penguin? Divergent population trajectories and the challenge of assessing the threat status of the Erect-crested penguin
Source: PLoS One. 2026 Oct 21;21(10):e0359075. doi: 10.1371/journal.pone.0359075 (PMC13686514; doi:10.1371/journal.pone.0359075)
Supplement: Supplementary file 4 [file pone.0359075.s005.docx]

We thank the three reviewers for their time and constructive comments, which have helped clarify and strengthen the manuscript throughout. Our point-by-point responses follow below, with reviewer comments in italic text and our responses indented beneath them.

### *Reviewers' comments:*

#### Reviewer #1: Review Schrödinger’s Penguin

*Topic is interesting and raises an important point for consideration and discussion. Writing is excellent. Methods are interesting and seem appropriate, I cannot evaluate the power of drone deploys ML models, but the extra work done to verify outcomes is convincing, especially with the inclusion of ground-truth data. Results and discussion are interesting and important. Overall, this paper makes fascinating and compelling argument for having a more nuanced IUCN assessment approach in such cases.*

*Some very minor points for editing:*

*Scientific names are not always italicised (e.g. line 154).*

*Line spacing changes at 715 – 760.*

*Line 290/291: wording is ambiguous “Erect-crested and Eastern Rockhopper penguins occasionally breed together” — could mean they breed within the same areas or actually mate with each other. Disambiguate.*

We are grateful to the reviewer for their generous and encouraging assessment of the manuscript, and for recognising the significance of the argument we set out to make regarding IUCN assessment approaches for species with divergent subpopulation trajectories. We also thank them for their careful eye in catching these minor but important editing points:

1. Scientific names not always italicised (e.g. line 154). Corrected throughout the manuscript.
2. Line spacing changes at 715-760. Corrected.
3. Line 290/291: We have revised this sentence to clarify that the two species breed in close proximity rather than interbreed: "Erect-crested and Eastern Rockhopper penguins occasionally breed in close proximity on the Antipodes Islands, but the DroneDeploy models did not distinguish between the two species."

#### Reviewer #2:

*This study investigates the population trajectory of the Erect-crested penguin, showcasing the divergent paths unfolding for the species at New Zealand’s Antipodes and Bounty Islands. The findings could justify removing the species from the “endangered” ranking on the IUCN Red List, bringing the authors to grapple with the validity of the Red List, and consider whether the real threats to the Erect-crested penguin can be so easily classified. These are things well worth considering, especially given the possibility of expanded fishing grounds that could threaten the species’ final stronghold.*

*The study is clear and well-written, and I just have minor suggestions:*

*Line 351 - 355 - Given how much nest density varied between islands (according to lines 403-405), is it reasonable to use the counts from Proclamation Island as a proxy for the whole of the Bounty Islands? Perhaps this can be justified in some way.*

We agree this warrants clarification and have added text to the Methods justifying Proclamation Island as a trend proxy. While absolute nest density varies among islands - likely reflecting differences in topography and space available among mixed-species colonies - there is no reason to expect the rate of change to differ between islands, as all are subject to the same broad-scale drivers.

*Line 289 - 290 - Why use the AI annotation if each point must be inspected and missed penguins added? No harm I suppose, I am just wondering if it makes the process faster.*

The AI annotation model substantially speeds up the process compared to fully manual annotation. However, it is important to note that the models were trained specifically on manual annotations from the Antipodes Islands, so manual verification of the resulting data served two purposes: improving the accuracy of the annotations themselves, and identifying false positives and false negatives to further refine the models. Ideally, this dual-purpose verification should be part of any AI-assisted annotation of survey data, since models will inevitably make mistakes. This is particularly feasible when the dataset size allows verification of the entire dataset, as was the case here with some ~38,000 annotations; this would clearly be different for species where colonies comprise millions of individuals.

*Line 322 - Is it possible to justify this assumption?*

This is the same underlying concern raised above regarding lines 351–355; please see our response there.

*Overall - It may be interesting to discuss more on alternatives to the current system of the IUCN Red List. How could it be improved to provide more accurate assessments of species at risk? I am sure there are many such cases of complicated population trajectories across different species, and improving the Red List criteria could therefore have many applications.*

We thank the reviewer for raising this important point, and agree that the paper benefits from further discussion of the Red List's capacity to handle divergent subpopulation trajectories. We have added a passage to the Discussion (“IUCN threat ranking conundrum” section) addressing this. We note that the IUCN guidelines already anticipate that global aggregation can mask regional crises, and provide a mechanism, the regional Red Listing, intended for such cases. However, this mechanism is designed primarily for species that are secure across most of their range but threatened locally, and offers less clear guidance for species like the Erect-crested penguin, whose entire global population is confined to a small number of comparably sized sites on opposing trajectories. We further highlight that the Red List's headline category, not its more nuanced supporting justification, is what is overwhelmingly consulted and acted upon by decision-makers. We do not believe this is a problem that can be solved by asking users to engage more closely with documentation they do not, in practice, consult. Rather, it points to a responsibility residing with the assessment and reporting system itself. While we consider the specific mechanism for achieving this to fall outside the scope of the present study, we believe highlighting the problem is a valuable contribution for the IUCN and wider conservation community to consider.

#### Reviewer #3:

*The manuscript is generally well written and presents a valuable reassessment of Erect-crested penguin population trajectories using improved drone-based surveys. I did not identify any major methodological flaws. The Discussion is somewhat long, but it is comprehensive and relevant.*

*My main comments concern the clarification of how drone-based penguin counts were converted to nest estimates (see detailed comments). In particular, in lines 428–433, although I could generally follow the rationale, the flow of this section is somewhat difficult to understand. I suggest clarifying, preferably using equations, which quantities were directly observed, which were estimated, and how the final estimates of total abundance and/or breeding population size were calculated. This is particularly important because the study does not apply a single uniform conversion across all breeding sites; rather, the approach used to derive population estimates differs among breeding locations. Clearly distinguishing these procedures would greatly improve the transparency and reproducibility of the estimates.*

This is a very valid point and we have revised the Results section accordingly. For the Bounty Islands, we now state explicitly that a fixed correction factor *r* = *N_g_* / *P_d,ref_*, from paired nest and drone counts on Proclamation Island) was applied uniformly across islands. For the Antipodes Islands, we clarify that the extended 50-day survey period required the nest-to-penguin ratio to instead be modelled as a linear function of survey date, applied per colony. We have stated explicitly that these two procedures differ structurally, reflecting the different survey durations and breeding-stage coverage at each site.

*In addition, I think the methods used by the authors are sustainable and less dependent on individual observer expertise, which appears to have been one of the limitations of historical surveys, especially on the Antipodes Islands. I therefore suggest that the authors more explicitly state the advantages of these methods, as well as what should be improved to achieve more accurate and efficient long-term monitoring.*

We agree that our approach offers a substantial advantage over historical survey methods in this respect. This is already discussed as such in the manuscript ('Divergent population trends’ section). Here we note that the reliability of the Bounty Islands time series rests on methodological consistency. This contrasts with the Antipodes Islands, where each historical survey employed different methods and levels of coverage, rendering direct comparisons uncertain.

Regarding further improvement, we have considered this carefully and believe the current approach is close to the practical limit achievable with existing technology. On the Bounty Islands, more efficient drones now allow the entire archipelago to be surveyed within a single day. However, since birds are largely sedentary during incubation, any gain in comparability from further reducing survey duration is likely marginal, as day-to-day and hour-to-hour fluctuation is related to non-breeders present. These numbers fluctuate between hours as they do between days which introduces comparable variability irrespective of survey speed.

On the Antipodes Islands, the primary constraint is the scale of the island and the exposure of colonies to prevailing weather, e.g. westerly conditions restrict surveying to the lee coast and vice versa. Such a limitation could not be overcome, for example, by using multiple drone teams simultaneously. Higher-resolution satellite imagery may eventually offer an alternative but remains similarly constrained by the region's persistent cloud cover. Consistency of survey timing relative to breeding stage is the most important factor for maximising comparability of estimates across future expeditions for the reasons outlined in the Methods ('Timing of the surveys').
